# Incidence of Spontaneous Pulmonary AVM Rupture in HHT Patients

**DOI:** 10.3390/jcm10204714

**Published:** 2021-10-14

**Authors:** Adam Fish, Katharine Henderson, Alex Moushey, Jeffrey Pollak, Todd Schlachter

**Affiliations:** Department of Interventional Radiology, Yale School of Medicine, New Haven, CT 06510, USA; adam.fish@yale.edu (A.F.); katharine.henderson@yale.edu (K.H.); alexander.moushey@yale.edu (A.M.); Jeffrey.Pollak@yale.edu (J.P.)

**Keywords:** hereditary hemorrhagic telangiectasia (HHT), pulmonary AVM, pulmonary AVM rupture

## Abstract

The spontaneous rupture of pulmonary AVMs, resulting in pulmonary hemorrhage and hydrothorax, is a life-threatening complication. While this phenomenon has been previously reported, the true incidence is not yet known. This study retrospectively reviewed records of 801 HHT patients with pulmonary AVMs to identify a single lifetime episode of hemothorax or pulmonary hemorrhage secondary to pulmonary AVM rupture. The lifetime prevalence and incidence of pulmonary AVM rupture in HHT patients was 2.7% and 0.16% respectively. In these patients, AVM rupture represented the initial presentation of HHT in nine (40.9%) cases and was life-threatening in nine (40.9%) cases. All cases occurred in virgin lesions, and subsequent embolization was curative. While a feared complication, pulmonary AVM rupture is rare and is likely effectively prevented by existing embolization techniques and indications.

## 1. Introduction

Hereditary hemorrhagic telangiectasia (HHT), also known as Rendu-Osler-Weber disease, is an autosomal dominant condition with an estimated prevalence of 1 in 5000, manifesting in mucosal telangiectasia and visceral telangiectasia and arteriovenous malformations [1,2,3]. Pulmonary arteriovenous malformations (PAVMs) are a feared manifestation of HHT. With vigorous screening of HHT patients, PAVM can be found in 23–61%, with a greater prevalence in type 1 than type 2 [4,5,6,7,8,9,10]. The familial form of PAVM is far more common than the sporadic form, with approximately 80–90% of patients with PAVM eventually displaying these lesions as part of the HHT-related clinical spectrum [11,12]. One of the most common and feared complications of PAVM includes paradoxical emboli, occurring in up to 30% of patients, which may result in stroke and brain abscess and, less commonly, systemic abscess [13]. Other complications include hypoxia, rupture, and high cardiac output, leading to heart failure and pulmonary hypertension [14].

Primary rupture of pulmonary AVMs resulting in pulmonary hemorrhage and hemothorax is a life-threatening complication that has been previously reported [15]. Pulmonary AVMs are thin-walled vessels that transmit high flow and are therefore theoretically prone to rupture. However, whether this is a practical or theoretical risk has yet to be determined; to our knowledge, the prevalence and incidence of spontaneous pulmonary AVM rupture in HHT patients is not yet known. Endovascular treatment remains the mainstay treatment of ruptured AVMs, though there is a paucity of large-volume studies in HHT patients. To our knowledge, this study represents the largest volume study of HHT patients with ruptured pulmonary AVMs.

## 2. Materials and Methods

### 2.1. Patient Selection

**Figure 1:**
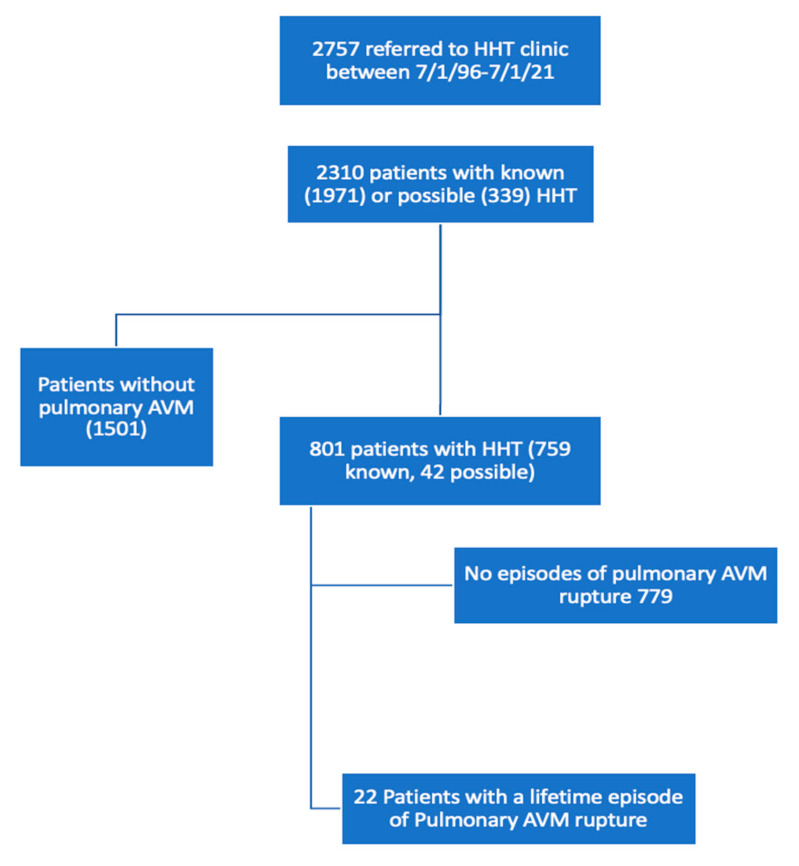
Flow chart.

A flow-chart demonstrating patient selection is shown in Figure 1. The records of the 801 patients with pulmonary AVMs and known or possible HHT were reviewed to identify a single lifetime event of clinically significant hemoptysis or hemothorax that led to a diagnosis of ruptured pulmonary AVM. All patients underwent screening for pulmonary AVMs, unless specifically referred for known pulmonary AVMs. In the 1990s, screening was performed with pulse oximetry and arterial blood gas sampling. This was then replaced with contrast echocardiography and chest CT. The diagnosis of primary ruptured AVM was made by CT showing lobar hemorrhage or hemothorax in the region of a pulmonary AVM when no other possible sources or causes could be identified. For patients with a history of pulmonary hemorrhage prior to being referred to our center, outside CT images and records were obtained to confirm the diagnosis of pulmonary hemorrhage secondary to AVM rupture. Previously embolized lesions were not excluded from the patient search, though all identified ruptured AVMs were found to have occurred in untreated or “virgin lesions”. Post-embolization hemoptysis occurring in the immediate post-procedural time period were not included, as this study looked at primary pulmonary AVM rupture. Confounding illnesses such as pulmonary embolism, pneumonia and acute heart failure were excluded.

### 2.2. Patient and Lesion Characterization

Records of the 22 patients with primary AVM rupture were then further evaluated for age, gender, HHT status (known vs. probable), family history, other AVMs, history of heart failure, cardiac disease, anti-coagulation use, lung disease, pregnancy, lesion location (of the ruptured AVM) and clinical presentation. Lesion location was divided into intra-parenchymal and subpleural. In eight cases, these events were documented as part of the patient’s remote history, prior to establishing care at our center, for which detailed angiographic and surgical records were unavailable. In four cases, an open surgical (non-endovascular) approach was taken (including two without detailed surgical records). Angiographic imaging and/or detailed angiographic dictations were available for 12 patients with primary pulmonary hemorrhage secondary to AVM rupture. In these patients, the diameter of the largest feeding artery and the AVM morphology (simple vs. complex) was recorded. Morphology was divided into simple-type (lesions with a single arterial feeder) and complex-type (lesions with 2 or more segmental pulmonary feeding arteries). The average, range and standard deviation were calculated.

### 2.3. Follow-Up

The average follow-up period, defined as the time between the initial and most recent encounters, was 16.3 years, with a range of 0–25 years. Patient records were searched for repeat episodes of pulmonary AVM rupture. Lesions were considered to be successfully treated if there were no further episodes of hemoptysis, hemothorax or pulmonary hemorrhage secondary to pulmonary AVM rupture.

## 3. Results

### 3.1. Incidence and Prevalence

Between 2 July 1996 and 22 July 2021, 2757 patients were referred to our HHT and Vascular Anomalies Clinic with concerns regarding HHT. During this time period, 1971 patients were diagnosed with known HHT and 339 patients with possible HHT according to the Curacao criteria [16] or by genetic testing. Records of the 2310 patients with known or possible HHT were reviewed for the presence of pulmonary AVMs. A total of 801 patients with known (759 patients) or possible (42 patients) HHT were identified as having pulmonary AVMs. Considering all pulmonary AVM patients referred to our clinic (930 patients), 759 (81.6%) were diagnosed with HHT and 42 with possible HHT (86.1% cumulative rate). Of the 801 patients with HHT or possible HHT, 22 patients had at least one lifetime episode of primary pulmonary hemorrhage resulting from a ruptured pulmonary AVM, up to the time of assessment. We therefore estimate a lifetime prevalence of 2.7% in HHT patients with pulmonary AVMs and 0.9% in all patients with known or possible HHT (or 1.1% in patients with known HHT). Of these 22 patients, 19 had known HHT and three were classified as possible HHT. At the time of assessment, the average follow-up period was 16.3 years, with a range of 0–25 years. We therefore estimate an annual incidence rate of pulmonary AVM rupture of 0.16% (22 cases/(801 patients x their age at most recent follow-up)) in HHT patients with pulmonary AVMs.

### 3.2. Patient Characteristics

**Figure 2:**
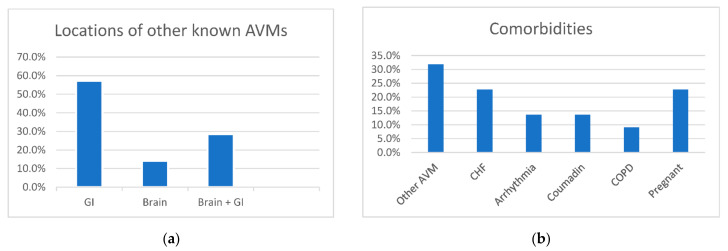
Bar graphs demonstrating the locations of other (**a**) AVMs and (**b**) co-morbidities in HHT patients with ruptured pulmonary AVMs. (AVM—arteriovenous malformations, CHF—chronic heart failure, COPD—chronic obstructive pulmonary disease, GI—Gastrointestinal).

Known or probable family history was identified in 19 patients (86.4%). Additional visceral AVMs were identified in seven patients (31.8%) (Figure 2a), chronic heart-failure in five (22.7%), arrhythmias in three (13.6%), anti-coagulation use in three (13.6%), and COPD in two patients (9.1%) (Figure 2b). There was a slight increase in pulmonary AVM hemorrhage seen in women (17 women out of 22 with hemorrhage). Whereas 67% (1548 patients) of the HHT patients seen in our clinic were women, 77% of patients diagnosed with AVM rupture were women. The prevalence ratio of women compared to men with pulmonary AVM hemorrhage was 1.08 (95% confidence interval of 0.76–1.56). Though not statistically significant, this is likely explained by an increased risk during pregnancy, as five patients (22.7%) were noted to be pregnant at the time of pulmonary hemorrhage. This is consistent with prior reports of increased risks for pulmonary hemorrhage during pregnancy [17].

### 3.3. Clinical Presentation

A breakdown of clinical presentation and lesion characterization is listed in Table 1. Hemoptysis was the most common presentation, seen in 64% of patients, with the remainder presenting with hemothorax (36%). Life-threatening instability occurred in nine patients (40.9%), which resulted from pulmonary hemorrhage in three cases (13.6%) and hemothorax in six cases (36.4%). As expected, all cases of pulmonary hemorrhage were caused by lobar lesions and all cases of hydrothorax were caused by subpleural lesions. In nine patients (40.9%), pulmonary AVM rupture represented the initial patient presentation, with a diagnosis of HHT subsequently obtained.

### 3.4. Lesion Characterization

**Figure 3:**
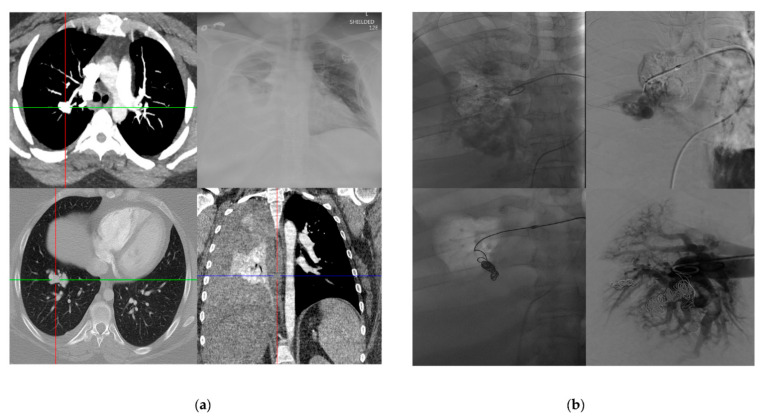
A 40-year-old female with HHT who presented with hemoptysis. (**a**) CXR showing large left-sided hemothorax, confirmed on CT, causing collapse of the adjacent lung. (**b**) Pulmonary angiography demonstrating a complex RLL AVM, followed by coil embolization and resolution of bleeding.

All cases of pulmonary AVM rupture occurred in virgin lesions (not previously treated). Complex lesions represented 50% of the cases. The size of the largest feeding pulmonary artery measured 7.1 mm +/− 2.9 (2.5–12). The majority of ruptured AVMs were identified in the lower lobes, 17 cases (77.2%). An example of a case of pulmonary AVM rupture is shown in Figure 3a,b.

### 3.5. Treatment Outcomes

Embolization was performed in 18 cases, lobectomy in four and chest tube placement in three cases. Embolization was nearly always curative (94.4%), with re-bleeding occurring in only one case. In the only case of re-bleeding, the degree of hemoptysis was minor, and angiography demonstrated new-feeding arteries into the nidus. The patient was subsequently re-treated, and no additional episodes of hemoptysis occurred.

## 4. Discussion

The risk of pulmonary AVM rupture in HHT patients is routinely listed in textbook chapters and papers. However, it was previously unclear whether this complication was a theoretical or clinically significant risk. To our knowledge, this study represents the largest single-institution retrospective review of ruptured pulmonary AVMs in HHT patients.

HHT is an autosomal dominant condition found in 1 in 5000 patients. Of the 2310 patients diagnosed with known or possible HHT at our clinic, 801 were found to have pulmonary AVMs (34.7%). This is in keeping with prior studies estimating 23–61% [4,5,6,7,8,9,10]. A total of 930 patients were referred for pulmonary AVMs, of whom 86.1% were diagnosed with known or possible HHT, which is similar to prior reports of 80–90% of all pulmonary AVMs [11,12] being secondary to HHT. Additional AVMs were identified in seven patients (31.8%), but this is likely an underestimation because, while we screen for brain AVMs, we do not routinely screen for extracranial AVMs.

After retrospectively reviewing 801 HHT patients with pulmonary AVMs (759 patients with known and 42 with possible HHT), a total of 22 patients were identified with a lifetime event of pulmonary AVM rupture. Clinical presentation included hemoptysis, pulmonary hemorrhage or hemothorax secondary to ruptured pulmonary AVMs. We therefore conclude a 2.7% lifetime prevalence and a 0.16% annual incidence of pulmonary AVM rupture in HHT patients. It is important to note that these figures represent the prevalence and incidence in HHT patients with pulmonary AVMs. When considering all HHT patients, the lifetime prevalence is 0.9%.

In addressing a few potential biases, it is important to note that because the hospital is a referral center for both HHT and pulmonary AVMs, it is possible that the true prevalence of pulmonary AVMs and thus pulmonary hemorrhage is lower than these estimates. However, given that the prevalence of pulmonary AVMs in our HHT population is within the previously reported range, these figures are likely to be fairly accurate. Furthermore, while eight patients in this study had episodes of pulmonary hemorrhage prior to referral to our center, these patients were not transferred for hemorrhage. Instead, due to relocating, they established care at our center years later.

Due to the small number of cases in this study over a 25-year period, it is difficult to draw firm conclusions about patient and lesion characterizations. Furthermore, of the 22 patients identified with pulmonary AVM rupture, angiographic imaging or detailed dictations were available for only 12 patients. Within these limitations, the ruptured AVMs identified were more complex, representing six (50%) of the cases, and larger, measuring 7.1mm +/- 2.9 (2.5–12), than the general HHT population. It should be noted that these six patients were previously included in a study describing patients with diffuse pulmonary AVMs [18]. Previous reports estimate that 20% of HHT pulmonary AVMs are complex lesions [11] and 60% of feeding arteries are between 3–5 mm [19]. A recent report from Ma et al. showed that pulmonary hemorrhage is more common in simple AVMs [15]. However, this study included patients without HHT. Furthermore, because the number of patients with pulmonary hemorrhage is small, these conclusions are limited.

Embolization proved to be highly effective in treating pulmonary AVM ruptures. In all but one case (94.4%), there were no instances of re-bleed. In this case (Table 1—Hemoptysis, Number 4), the re-bleed was due to pulmonary artery collateral inflow. The resultant hemoptysis was clinically insignificant and was resolved with embolization. It is interesting to note that of the 22 patients who presented with pulmonary AVM rupture, nine cases represented the initial presentation. Therefore, when considering HHT patients who are followed, the cases of rupture become even more rare. Furthermore, all cases of AVM rupture (100%) occurred in virgin lesions. These factors strongly suggest that current embolization guidelines are effective in preventing pulmonary rupture, and they also emphasize the importance of HHT screening and the harm caused by diagnostic delays in patients with HHT [20]. In terms of current HHT patient management, it is worth noting that at this institution, all pulmonary AVMs that can be accessed are embolized.

## 5. Conclusions

While pulmonary AVM rupture is indeed a real and serious complication of HHT, its lifetime prevalence and incidence (2.7% and 0.16%, respectively) are rare. Embolization is highly effective in both treating and preventing pulmonary AVM rupture, as demonstrated by the fact that all cases of rupture (100%) occurred in virgin lesions, and only one case of clinically insignificant re-bleeding (5.6%) occurred following embolization.

## Figures and Tables

**Table 1:** Patient characteristics.

Characteristics of Detected PAVMs											
Patient	Age (Study)	Age (Rupture)	Sex	HHT	Family Hx of HHT	Lobe	Location	Type (Simple/Complex)	Largest Feeding Artery Diameter (mm)	Life-Threatening	Re-Bleed Following Embolization
Hemothorax											
1	72	39	F	Definite	Known	LLL	Subpleural	Non-angiographic treatment	Yes	No	
2	58	57	F	Definite	Known	RLL	Subpleural	Complex	9	Yes	No
3	57	20	F	Definite	Known	RLL	Subpleural	Records/Imaging unavailable	Yes	No	
4	62	43	F	Definite	Known	LLL	Subpleural	Complex	10	Yes	No
5	72	57	F	Definite	Known	LLL	Subpleural	Records/Imaging unavailable	Yes	No	
6	39	24	F	Definite	Known	LLL	Subpleural	Records/Imaging unavailable	No	No	
7	41	38	F	Definite	Known	RML	Subpleural	Complex	7	No	No
8	54	43	M	Definite	Known	RLL	Subpleural	Simple	6	Yes	No
Hemoptysis											
1	58	26	F	Definite	Known	LLL	Lobar	Complex	11	No	No
2	65	60	F	Definite	Known	LLL	Lobar	Simple	4	No	No
3	47	42	F	Definite	Known	LLL	Lobar	Simple	2.5	No	No
4	71	69	F	Possible	None	RUL	Lobar	Complex	12	No	Yes
5	66	59	F	Definite	Known	RUL	Lobar	Records/Imaging unavailable	Yes	No	
6	74	28	F	Definite	Unknown	RLL	Subpleural	Simple	7.5	No	No
7	44	23	F	Definite	Known	RLL	Lobar	Non-angiographic treatment	Yes	No	
8	48	23	F	Definite	Known	LLL	Lobar	Non-angiographic treatment	Yes	No	
9	31	28	F	Definite	Known	LLL	Lobar	Records/Imaging unavailable	No	No	
10	20	18	F	Definite	Probable	RLL	Lobar	Complex	6	No	No
11	79	78	M	Definite	Known	RUL	Lobar	Records/Imaging unavailable	No	No	
12	30	25	M	Possible	Probable	LLL	Lobar	Non-angiographic treatment	No	No	
13	75	74	M	Definite	Known	RLL	Lobar	Simple	5	No	No
14	89	89	M	Possible	None	LUL	Lobar	Simple	5.5	No	No

HHT, Hereditary hemorrhagic telangiectasia; PAVMs, Pulmonary arteriovenous malformations; LLL—Left lower lobe; RLL—Right lower lobe; RML—Right middle lobe; RUL—Right upper lobe; LUL—Left upper lobe.
